# Punch Excision Combined With Radiotherapy for Keloid Treatment

**DOI:** 10.1111/jocd.70764

**Published:** 2026-02-22

**Authors:** Yunfeng Guan, Meiling Huang

**Affiliations:** ^1^ Huzhou Central Hospital Fifth School of Clinical Medicine of Zhejiang Chinese Medical University Huzhou China

**Keywords:** adverse effects, keloid, punch excision, radiotherapy, treatment outcome

## Abstract

**Background:**

Punch excision is gaining recognition in keloid management. Its distinct mechanism from conventional surgery necessitates a re‐evaluation of the efficacy and safety when combined with radiotherapy (RT), an area currently lacking evidence.

**Aims:**

To investigate the clinical efficacy and safety of punch excision combined with RT for treating trunk keloids and to compare the differences between perioperative (RT initiated one day before surgery) and postoperative RT (RT within 24 h after surgery) regimens.

**Methods:**

A retrospective study was conducted on patients with trunk keloids treated with punch excision combined with electron beam RT from January 2022 to January 2024. Outcomes were assessed using the Patient and Observer Scar Assessment Scale (POSAS) and records of adverse reactions.

**Results:**

The study included 34 patients. At 18 months, all keloids improved significantly without recurrence. The total Observer Scale score decreased by 52.73% and the Patient Scale score by 62.77%. No significant difference in POSAS scores was found between the two groups at 18 months. The perioperative group showed advantages in shorter postoperative bleeding duration and lower incidence of moderate‐to‐severe acute pain.

**Conclusion:**

Punch excision combined with RT is effective for trunk keloids. While long‐term cosmetic outcomes are similar between perioperative and postoperative RT, the perioperative regimen offers benefits in reducing early postoperative complications.

## 1 Introduction

Keloids are dermal fibroproliferative disorders characterized by elevated levels of interleukin‐6 and transforming growth factor‐beta [1, 2], which can cause pain, pruritus, and significantly reduce patients' quality of life [3]. When located on exposed areas, they can also lead to cosmetic concerns and psychological distress. Among existing treatment modalities, punch excision is increasingly becoming an effective option for keloid management due to its simplicity and minimal invasiveness [4]. Notably, unlike traditional wide excision, punch excision removes the lesion via small, precise circular cuts, preserving surrounding normal tissue to the greatest extent but leaving behind small amounts of scar tissue. These differences suggest that conclusions regarding timing, dosage, and efficacy from previous adjuvant radiotherapy (RT) strategies based on traditional excision may not be fully applicable to punch excision. Currently, clinical studies specifically focused on punch excision combined with RT for keloids remain limited. Although a recent study has reported promising outcomes using punch excision combined with superficial X‐ray therapy (a form of RT), evidence regarding its combination with electron beam RT and comparisons between different RT timing strategies is still scarce [5]. Therefore, this study aims to systematically evaluate the clinical value of punch excision combined with electron beam RT and to compare the efficacy differences between perioperative and postoperative RT regimens.

## 2 Materials and Methods

### 2.1 Clinical Data

A retrospective analysis was performed on data from all patients with trunk keloids who underwent punch excision combined with RT at our institution between January 2022 and January 2024.

### 2.2 Inclusion and Exclusion Criteria

Inclusion criteria were as follows: (1) patients diagnosed with trunk keloids by dermatology specialists; (2) aged over 18 years; (3) who underwent keloid punch excision and adjuvant electron beam RT. Exclusion criteria were: (1) receipt of any anti‐keloid treatments either preoperatively or during the 18‐month postoperative follow‐up period; (2) inability to cooperate with the complete treatment and follow‐up process; (3) keloids accompanied by infection or significant contracture.

Data were collected from patient records using standardized forms. Basic patient information included age, gender, keloid location, size, duration, and family history.

### 2.3 Treatment

#### 2.3.1 Punch Excision Surgery

Under local anesthesia (2% lidocaine mixed with 1:100 000 epinephrine for analgesia and hemostasis), a sterile punch with a diameter of 2–4 mm was selected based on our clinical experience with keloid size and thickness: typically, a 2‐mm punch for small lesions (< 3 cm^2^ or ≤ 3 mm thick), a 3‐mm punch for medium‐sized keloids (3–8 cm^2^ or 3–5 mm thick), and a 4‐mm punch for large lesions (> 8 cm^2^ or > 5 mm thick). This selection was guided by clinical judgment to balance effective debulking and dermal bridge integrity preservation, with no rigid or universal cutoffs.

A central vertical incision was first made at the keloid site, targeting the lesion's central proliferative core. Subsequently, using this central hole as the origin, multiple radial punch excisions were performed outward—either vertically or at an appropriate angle determined by lesion morphology and surgical field visibility—with an inter‐hole spacing of 3 mm in most cases. The procedural endpoint of punch excision was set to maximize scar tissue debulking while ensuring the exposure of normal‐colored, vascularized subcutaneous fat and the preservation of intact dermal bridges between adjacent punch holes; this endpoint was designed to prevent ischemia‐induced necrosis and facilitate subsequent wound healing. After each punch pass, residual dense scar tissue was meticulously excavated layer by layer with curettes to ensure thorough clearance of fibrotic tissue within the lesion boundary.

For exceptionally hypertrophic keloids or those with localized firm nodules that could not be adequately debulked by punch excision alone, targeted surgical excision or keloid core excision was performed as an adjunctive procedure. In all cases, punch excision constituted the primary intervention, responsible for the overwhelming majority (> 80%) of tissue removal. Any additional excisions were minor and localized, and thus did not change the procedure's essential character as punch‐based.

#### 2.3.2 Radiotherapy

**FIGURE 1:**
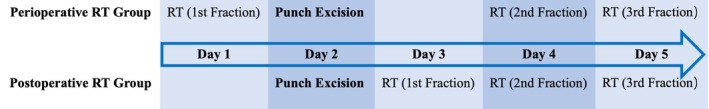
Radiotherapy schedules for the two groups.

RT was delivered using a linear accelerator with 4–8 MeV electron beams. The radiation field encompassed the lesion area with a 1.0 cm margin of normal tissue. A 0.5 cm bolus was applied to the skin surface throughout the treatment. A total dose of 18 Gy was administered in three daily fractions. Depending on the timing of RT, two regimens were employed: the perioperative group received the first fraction one day before surgery, followed by the second fraction within 24–48 h after surgery, and the third fraction on the subsequent day; the postoperative group commenced RT within 24 h after surgery, completing the total dose of 18 Gy in three once‐daily fractions. A schematic diagram of the RT timing is shown in Figure 1.

### 2.4 Observation Indicators

The efficacy of punch excision was assessed over an 18‐month follow‐up period. The Patient and Observer Scar Assessment Scale (POSAS 2.0) was used for comprehensive evaluation. Observers quantified the microscopic qualities of the scar (Vascularity, Pigmentation, Thickness, Relief, Pliability, Surface Area), while patients reported subjective sensations (Pain, Itch, Color, Stiffness, Thickness, Irregularity). For patients with multiple keloids, one ‘target keloid’ was designated for evaluation. Selection criteria were: (1) the lesion with the highest total baseline POSAS 2.0 Observer Scale score; (2) if scores were equal, the lesion with the largest area was selected. Only data from this target keloid per patient were included in the statistical analysis. Adverse reactions were monitored and recorded throughout the study.

### 2.5 Data Analysis

All statistical analyses were performed using SPSS 26.0 software. Continuous variables conforming to a normal distribution are presented as mean ± standard deviation (or 95% confidence interval), while those not conforming are presented as median (interquartile range). Categorical variables are described as frequency (percentage). Comparisons of baseline characteristics and adverse reactions between groups were performed using independent samples *t*‐test or Mann–Whitney *U* test for continuous variables, and chi‐square test or Fisher's exact test for categorical variables. Paired sample *t*‐test (or Wilcoxon signed‐rank test) was used to analyze changes in POSAS 2.0 scores from baseline to 18 months postoperatively for all patients. Analysis of covariance (ANCOVA) was used for intergroup efficacy comparison, with the 18‐month postoperative score as the dependent variable, treatment group as the fixed factor, and the corresponding baseline score as the covariate for adjustment. All hypothesis tests were two‐sided, with a *p*‐value < 0.05 considered statistically significant.

## 3 Results

A total of 34 patients were included in this study. Among these, 4 patients required minor adjunctive surgical excision as described in Section 2. The remaining 30 patients underwent punch excision alone. All patients completed the designated treatment plan and the full follow‐up. According to the radiotherapy timing, patients were divided into the perioperative RT group (*n* = 13) and the postoperative RT group (*n* = 21). The baseline characteristics of these two groups were comparable (Table 1).

**TABLE 1:** Patient characteristics.

Characteristic	Perioperative RT group (*n* = 13)	Postoperative RT group (*n* = 21)	*p*
Age, median (IQR)	29 (18.50)	37 (21.50)	0.17
Sex, *n* (%)			> 0.99
Male	4 (30.77)	7 (33.33)	
Female	9 (69.23)	14 (66.67)	
Site of keloids, *n* (%)			0.80
High‐tension areas^a^	8 (61.54)	12 (57.14)	
Other areas	5 (38.46)	9 (42.86)	
Duration (years), median (IQR)	5 (6.5)	3 (5.5)	0.26
Size (cm^2^), median (IQR)	6 (7.5)	8 (8)	0.86
Family history, *n* (%)			0.54
Yes	2 (15.38)	1 (4.76)	
No	11 (84.62)	20 (95.23)	
Minor adjunctive surgical excision,*n*(%)	1 (7.69)	3 (14.29)	> 0.99

^a^ Scars located in high‐tension areas (e.g., pre‐sternum, joints, shoulders, suprapubic) were compared to scars in other anatomical locations.

Efficacy evaluation showed that at 18 months postoperatively, scar condition had significantly improved in all patients, with no disease progression observed. The total POSAS 2.0 Observer Scale score and total Patient Scale score decreased by more than 30% for every patient, with average reductions of 52.73% (95% CI: 47.81%–57.65%) and 62.77% (95% CI: 58.15%–67.40%), respectively. To address potential confounding from adjunctive excision, a sensitivity analysis was performed on the subset of patients who underwent punch excision alone (*n* = 30): the average reductions were 52.36% (95% CI: 47.80%–57.06%) for the Observer Scale and 61.62% (95% CI: 56.66%–66.54%) for the Patient Scale, indicating a comparable magnitude of improvement.

**FIGURE 2:**
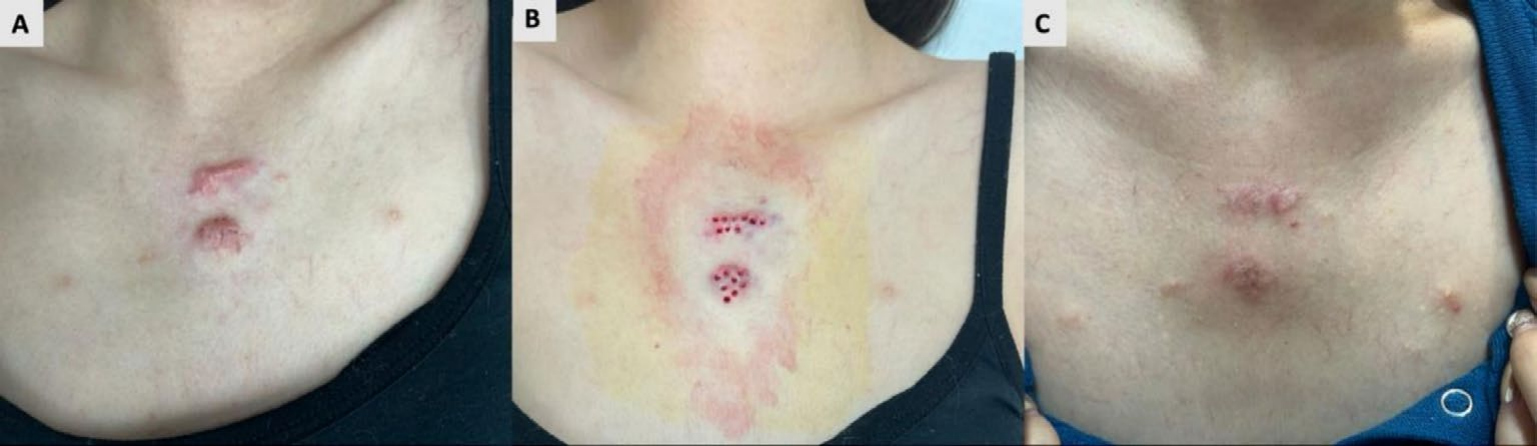
(A) A 36‐year‐old female patient presented with multiple keloids on the anterior chest wall secondary to acne excoriée, accompanied by pruritus and pain. (B) The patient underwent punch excision of the keloids, followed by adjuvant RT initiated the day after surgery, with a total dose of 18 Gy delivered in three fractions. (C) The 18‐month postoperative assessment revealed minimal residual scarring. The patient reported complete resolution of pain and pruritus and expressed high satisfaction with the aesthetic outcome.

**FIGURE 3:**
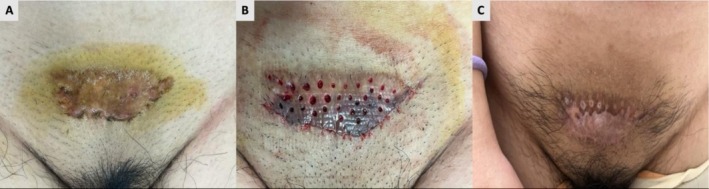
(A) A 24‐year‐old female patient presented with a suprapubic keloid secondary to trauma, accompanied by pain. (B) The treatment team first administered a 6–Gy radiation fraction. Punch excision, supplemented by core excision of a firm marginal nodule, was performed one day later. The remaining two fractions (6 Gy each) were completed starting on the second postoperative day. (C) The 18‐month follow‐up assessment showed near‐complete resolution of the original keloid and complete disappearance of pain. Although partial depigmentation occurred, the patient expressed high satisfaction with the overall treatment outcome.

After adjusting for baseline scores, no statistically significant differences were found in the total POSAS 2.0 Observer Scale scores or total Patient Scale scores at 18 months postoperatively between the perioperative and postoperative RT groups (Table 2). Figures 2 and 3 show representative images of two patients before, during, and after treatment follow‐up.

**TABLE 2:** Comparison of POSAS scores at 18 months (ANCOVA).

Outcome measure	Perioperative RT group (*n* = 13)		Postoperative RT group (*n* = 21)		
	Unadjusted mean (95% CI)	Adjusted mean (95% CI)	Unadjusted mean (95% CI)	Adjusted mean (95% CI)	*p*
POSAS Observer Scale	19.69 (15.94–23.45)	19.54 (15.92–23.16)	22.05 (19.21–24.88)	22.04 (19.21–24.89)	0.49
POSAS Patient Scale	17.69 (14.43–20.95)	17.66 (14.22–21.11)	16.76 (13.67–19.86)	17.10 (14.39–19.80)	0.15

Safety analysis indicated that the perioperative RT group had advantages in terms of postoperative bleeding duration and the incidence of moderate‐to‐severe acute pain. However, there were no significant differences between the two groups in postoperative bleeding rate, telangiectasia, or pigmentation abnormality (Table 3).

**TABLE 3:** Adverse reactions.

Adverse reactions	Perioperative RT group(*n* = 13)	Postoperative RT group (*n* = 21)	*p*
Bleeding, *n*(%)	11 (84.62)	20 (95.24)	0.54
Bleeding times (days), mean (SD)	1.08 (0.49)	1.67 (0.85)	0.03
Pain, *n* (%)	—	—	0.04
Mild	9 (69.23)	7 (33.33)	
Moderate to severe	4 (30.77)	14 (66.67)	
Dyspigmentation, *n* (%)	3 (23.08)	7 (33.33)	0.70
Telangiectasia, *n* (%)	1 (7.69)	1 (4.76)	0.72

## 4 Discussion

Punch excision has emerged as a core minimally invasive technique in keloid management. Its principle of removing scar tissue through multiple small‐aperture excisions preserves the surrounding epidermis and dermal bridges, thereby minimizing wound tension—a key promoter of keloid recurrence [6, 7]. This technique forms the basis for various effective combination therapies, such as with intralesional corticosteroids [8, 9, 10] or photodynamic therapy [11]. Notably, its combination with RT represents a promising strategy that merges mechanical decompression with potent anti‐proliferative action. Preliminary evidence, including a recent study utilizing punch excision combined with superficial X‐ray therapy, supports the efficacy of this approach [5]. Nonetheless, robust clinical evidence specifically evaluating its combination with electron beam RT remains limited, warranting further investigation.

Surgery combined with RT has been widely proven to be an effective strategy for treating refractory keloids, with recurrence rates controlled between 10% and 20% [12, 13]. In recent years, adjuvant RT protocols have been continuously optimized, with the timing of RT being a key factor influencing efficacy. Studies have shown that initiating RT within 24 h postoperatively can effectively inhibit fibroblast migration and proliferation, thereby significantly reducing the risk of recurrence [14]. Furthermore, perioperative RT (also known as “sandwich radiotherapy”) has also shown good results in traditional scar excision, potentially allowing more precise targeting of proliferating cells and reducing exposure to normal tissue through pre‐ and postoperative fractionated irradiation [15]. Regarding dosage, a biologically effective dose ≥ 30 Gy is considered a key threshold for controlling recurrence [16]; hypofractionation can achieve local control rates comparable to or superior to conventional fractionation without increasing complications [17, 18, 19].

Based on this background, our study specifically evaluated the combination of punch excision with electron beam RT, adopting a hypofractionated regimen (18 Gy in 3 fractions) and comparing two timing strategies: perioperative and postoperative RT. Efficacy evaluation showed that at 18 months postoperatively, all patients' keloid conditions had significantly improved, with POSAS 2.0 total score reduction rates exceeding 50% and no cases of disease progression, suggesting good feasibility and preliminary efficacy of this combination. Further analysis found that after adjusting for baseline scores, there were no statistically significant differences in the total POSAS 2.0 Observer and Patient Scale scores at 18 months postoperatively between the perioperative and postoperative RT groups, indicating comparable long‐term cosmetic outcomes between the two RT timing strategies.

The promising efficacy observed may be underpinned by a synergistic mechanism that concurrently addresses mechanical, cellular, and microenvironmental facets of keloid pathogenesis. First, punch excision transforms a rigid, hyper‐tense keloid plaque into a three‐dimensional mesh. This architectural change is hypothesized to achieve immediate and multidirectional release of internal mechanical tension, a factor critically implicated in keloid growth and persistence [6, 7]. Second, this created porous network likely alters the local tissue environment in favor of adjuvant RT. The multiple deep channels may facilitate more uniform penetration and distribution of electron beams compared to a flat wound bed after wide excision, potentially improving the targeting of residual fibroblasts within the dermal scaffold. This aligns with the principle that timely RT inhibits fibroblast proliferation [14], and the unique wound geometry may enhance this effect. Third, the preserved islands of epidermis and adnexal structures within the mesh could act as reservoirs for keratinocyte and stem cell migration, promoting faster re‐epithelialization over the punched defects. Accelerated wound closure is theorized to shorten the vulnerable inflammatory phase, a window during which pro‐fibrotic signaling is heightened [20, 21]. Thus, the combination of punch excision and RT constitutes a multi‐faceted approach. Punch excision provides mechanical decompression and creates a wound architecture that may facilitate RT delivery, while RT directly targets proliferative fibroblasts to prevent recurrence. Together, they address the biomechanical and cellular drivers of keloid pathogenesis synergistically.

Regarding safety, the perioperative RT group showed advantages in postoperative bleeding duration and the incidence of moderate‐to‐severe acute pain. This may be attributed to the temporal distribution of the RT course. In the perioperative group, RT was administered over approximately 5 days (one fraction preoperatively and two postoperatively), whereas the postoperative group received all three fractions in a concentrated manner within the first 3 days after surgery. This distributed schedule likely prevents the peak RT‐related tissue response (e.g., edema, inflammation) from directly coinciding with the peak of surgical trauma and pain in the immediate postoperative period. Furthermore, by avoiding RT‐related procedures, such as bolus application, within the first 24 h after surgery—a critical window for bleeding and acute pain—the perioperative regimen may mitigate additional mechanical and inflammatory stimuli during this vulnerable phase. These factors collectively reflect the comprehensive advantages of the perioperative strategy in optimizing the early postoperative recovery phase.

Despite the advantages of punch excision, such as minimal invasiveness and rapid recovery, the technique still has certain limitations. In cases of thicker keloids, punch excision may fail to completely remove the deep proliferative core, necessitating conversion to traditional excision^6^ or combination with keloid core removal surgery. Indeed, in our cohort, a minority of patients required such adjunctive focal excision for persistent nodules, reflecting the pragmatic clinical adaptation when pure punch excision is insufficient for the most hypertrophic components. To evaluate whether this introduced confounding, a sensitivity analysis was conducted on the subgroup undergoing punch excision alone (*n* = 30). The results demonstrated a closely similar magnitude of scar improvement in this subgroup, suggesting that the inclusion of cases with adjunctive excision did not substantially alter the primary efficacy conclusions of the study. Furthermore, existing relevant studies, including this one, primarily focus on Asian populations and are constrained by limited sample sizes, short follow‐up periods, and a lack of long‐term recurrence data. Future high‐quality, multi‐center, long‐term follow‐up studies are needed to further validate its efficacy and safety.

## 5 Conclusion

Punch excision combined with adjuvant radiotherapy is an effective and safe minimally invasive treatment regimen for trunk keloids, capable of significantly improving scar appearance and patient‐reported symptoms. Regarding the choice of RT timing, perioperative RT and postoperative RT yield equivalent cosmetic outcomes at 18 months postoperatively, but perioperative RT demonstrates advantages in reducing postoperative bleeding duration and the incidence of moderate‐to‐severe acute pain, indicating better postoperative recovery characteristics.

## Ethics Statement

This study was approved by the Ethics Committee of Huzhou Central Hospital (approval no.: 2025247‐01; Date of Approval: September 20th, 2025) and conducted in accordance with the ethical standards of the Declaration of Helsinki.

## Consent

Written informed consent was obtained from all participants for the publication of their anonymized clinical data and photographs.

## Conflicts of Interest

The authors declare no conflicts of interest.

## Data Availability

The data that support the findings of this study are available from the corresponding author upon reasonable request.
